# DENV2 Pseudoviral Particles with Unprocessed Capsid Protein Are Assembled and Infectious

**DOI:** 10.3390/v12010027

**Published:** 2019-12-25

**Authors:** Jyoti Rana, Oscar R. Burrone

**Affiliations:** Molecular Immunology Group, International Centre for Genetic Engineering and Biotechnology, ICGEB, Padriciano 99, 34149 Trieste, Italy

**Keywords:** dengue virus, capsid anchor, sequential cleavage, infectivity

## Abstract

Proteolytic processing of flavivirus polyprotein is a uniquely controlled process. To date, the sequential cleavage of the capsid anchor sequence at the junction of C-PrM has been considered essential for the production of flaviviruses. In this study, we used two experimental approaches to show the effect of unprocessed capsid on the production and infectivity of dengue virus 2 (DENV2) pseudoviral particles. The results showed that (1) both mature and unprocessed capsids of DENV2 were equally efficient in the viral RNA packaging and also in the assembly of infective particles; (2) DENV2 variants, in which the viral and host mediated cleavage of Ca peptide were independent, produced significantly higher levels of infective particles. Overall, this study demonstrated that unlike other flaviviruses, DENV2 capsid does not require a cleavable Ca sequence, and the sequential cleavage is not an obligatory requirement for the morphogenesis of infective pseudoviral particles.

## 1. Introduction

Dengue virus (DENV), a member of genus *Flavivirus* and family *Flaviviridae*, is one of the major emerging arthropod-borne pathogens infecting millions of people worldwide. Dengue virus has four distinct serotypes, and all of them can cause disease symptoms ranging from self-limited febrile illness, called dengue fever (DF), to dengue haemorrhagic fever (DHF) and dengue shock syndrome (DSS) [1]. Like a typical flavivirus, DENV is an enveloped virus having a single copy of positive sense RNA genome of approximately 11 kb in length. The viral genome encodes for a single polyprotein, which traverses the endoplasmic reticulum (ER) membrane multiple times and is co- and post-translationally processed into 10 viral proteins by viral and host proteases [2]. The structural proteins, capsid (C), membrane precursor (PrM) and the envelope protein (E) are located in the NH2-terminal, one-quarter of the polyprotein, followed by the non-structural proteins (NS). The virus-encoded serine protease (NS2B/NS3) and the cellular signal peptidase catalyse cleavages of the polyprotein precursor in the cytosolic side and in the lumen of the ER, respectively. Another protease, putatively located inside the ER, is required for processing at the carboxy terminus of the flavivirus protein NS1 [3,4]. Mature virions contain multiple copies of the C protein encapsulating the RNA genome to form the viral nucleocapsid [5,6,7] which is surrounded by a host cell lipid bilayer derived from the ER, embedded with 180 copies of M and E proteins.

Among all flaviviruses, the mature protein C is separated from the PrM by a hydrophobic signal sequence called capsid anchor (Ca) that serves as a signal peptide for translocation of PrM into the ER lumen. Cleavage by the luminal signal peptidase occurs efficiently only after upstream cleavage at the Ca N-terminus by the viral protease [8,9,10,11]. The sequential behaviour of these cleavages at the junction of C-PrM has been described as a critically important step in flavivirus assembly [12,13,14]. Although the capsid anchor of flaviviruses share common features, our previous studies have described that its involvement in the assembly and secretion of infective particles varies among viruses [15,16]. This encouraged us to study the effect of disruption of the sequential Ca cleavage on DENV2 production. Here, we describe the impact of unprocessed capsid and its sequential cleavage on the production of DENV2-infective pseudoviral particles using tripartite and bipartite complementation approaches.

## 2. Materials and Methods

### 2.1. Cell Lines, Monoclonal Antibodies and Chemicals

The HEK293T (ATCC CRL-11268, Rockville, MD, USA), Vero (ATCC CCL-81) and HeLa (ATCC CCL-2) cells were cultured in DMEM (Life Technologies, Paisley, UK) supplemented with 10% heat-inactivated foetal calf serum (FCS) (Life Technologies), 50 µg/mL gentamycin and 2 mMl-glutamine. Cells were incubated at 37 °C in the presence of 5% CO_2_. Flavivirus E protein-specific mAb 4G2 was expressed as a scFv [17] (NCBI accession codes: KJ438784 and KJ438785) fused to avidin in an avibody format and used as cultured supernatant from HEK293T transfected cells. The mAbs anti-SV5 and anti-Ro tag were obtained as previously described [18,19]. Anti-DENV2 capsid hybridoma cell line was kindly provided by John Aaskov, and culture supernatants were used for Western analysis [20].

### 2.2. Plasmid DNA Constructs

The gene fragment encoding for the *structural proteins* (C-PrM-E) from DENV2 NGC strain (GenBank accession number AAA42941) was obtained as a synthetic, mammalian-codon optimised gene (GenScript, Piscataway, NJ, USA) and cloned into pVAX1 expression vector (Life Technologies). The DNA-launched WNV sub-genomic replicon expressing EGFP (herein named WNV-rep) was kindly provided by Theodore Pierson (National Institute of Allergies and Infectious Diseases, MD, USA) [21]. The capsid constructs were obtained by cloning, while mutant and tagged packaging constructs were obtained by site-directed mutagenesis (QuikChange XL Site-Directed Mutagenesis Kit, Agilent Technologies, La Jolla, CA, USA) following manufacturer instructions. The Ro-tag tagged DENV2 NS2B/NS3 was synthesized and cloned in pcDNA3.1 vector (GenScript Corp.).

### 2.3. Production of Pseudoviral Particles

The DENV2 pseudoviral particles were produced using tripartite or bipartite as previously described [22]. Briefly, for the tripartite approach, WNV-rep, PrME and C gene constructs were used in a 1:1:4 ratio, respectively, for a bipartite approach, the WNV-rep and plasmids expressing the wild-type (wt) or mutant packaging constructs of DENV2 were co-transfected into HEK293T cells in a 1:3 ratio (WNV-rep:packaging construct ratio) using linear polyethylenimine (PEI, 1:3 DNA–polymer ratio, MW 25,000, Polysciences, Warrington, PA, USA). A 16 h post-transfection culture medium was replaced by DMEM with 7% FCS and pseudoviruses were harvested after 24 h of incubation at 37 °C. Supernatants were clarified by centrifugation and stored at −20 °C until use. When needed, the culture supernatant was ultra-centrifuged using 20% sucrose cushion with Beckman Coulter Airfuge centrifuge using an A100/18 rotor at 149,000× *g* for 2 h at 4 °C, and the pellet containing pseudoviruses was resuspended in non-reducing sample buffer for Western blot analysis.

### 2.4. Infection of Vero Cells

A total of 4 × 10^4^ Vero cells were seeded in 24 multi-well plates 24 h before infection. Culture media were removed and cells were infected with 200 µL of pseudoviral preparations for 3 h at 37 °C. Then, 0.5 mL of DMEM with 2% FBS was added, and cells were cultured for 24 h. Cell infection was determined by measuring the percentage of EGFP positive cells in cytofluorimetric analysis in a FACS Calibur (BD Biosciences, San Jose, CA, USA).

### 2.5. RT-PCR and Q-PCR

The WNV replicon-derived RNA was isolated from 100 µL of pseudoviral preparations using the RNAzol-BEE solution (Tel-Test, Friendswood, TX, USA) and then treated with DNaseI (Promega, Madison, WI, USA) following the manufacturer’s instructions. Reverse transcription was performed using random hexamers (Sigma) and M-MLV Reverse transcriptase (Life Technologies) according to the manufacturer’s protocol. The cDNAs were then amplified by PCR using WNV-3′UTR specific primers (Forward, 5′-CAGTGTCAGACCACACTTTAATGT-3′; Reverse, 5′-GCTTACAGCTTCAGCCAAG-3′). Real-time PCR was performed using Evagreen according to the manufacturer’s protocol.

### 2.6. Cleavage Analysis

The tagged version of either wt or mutant DENV2 packaging constructs were transfected in HEK293T cells in the presence or absence of WNV-rep or DENV2 protease using standard calcium phosphate protocol in 6multi-well plates as previously described [23]. After overnight incubation at 37 °C, culture medium was replaced with serum-free DMEM and cells incubated for another 24 h at 37 °C. The cellular extracts were prepared in 100 µL of TNN lysis buffer (100 mM Tris-HCl, pH 8, 250 mM NaCl, 0.5% NP-40) supplemented with Protease Inhibitor Cocktail (Sigma–Aldrich, St. Louis, MO, USA). Samples were stored at −20 °C until use.

### 2.7. Membrane-Associated Proteins Assay

Purified pseudo infective particles were resuspended in 0.1M Na_2_CO_3_ and incubated on ice for 15 min, followed by centrifugation at 52,000 rpm for 15 min. The pellet was resuspended in PAGE sample buffer, while the supernatant was treated with TCA for precipitation of soluble proteins. Briefly, TCA was added to supernatants (20% final), mixed, incubated on ice for 10 min and centrifuged at 14,000 rpm for 10 min. The pellet was washed with chilled acetone, dried and resuspended in PAGE sample buffer. Samples were then analysed by Western blot.

### 2.8. Western Blots

Western blot analysis of untagged, SV5- and HA-tagged proteins were done as previously described [18]. Briefly, samples were separated by non-reducing SDS-PAGE, transferred to polyvinylidenedifluoride (PVDF) membranes (Millipore, Temecula, CA, USA) and blocked with 5% milk solution in PBS (PBS-milk). For capsid, SV5- or HA-tagged proteins, membranes were incubated for 1 h with anti-C hybridoma supernatant, anti-SV5 mAb (1 µg/mL) or anti-HA (Sigma-Aldrich, 1:2000), washed and further probed with HRP-linked anti-mouse IgG goat antibodies (KPL, Gaithersburg, MA, USA, 074-1809, 1:10,000) for 1 h. In the case of 4G2, the avibody was previously conjugated with biotinylated-HRP (ThermoFisher-Pierce, Rockford, IL, USA), membrane was incubated for 1 h and washed. Mouse HRP-conjugated anti-actin (clone AC-15, Sigma–Aldrich, 1:30,000) was used as loading control. Blots were developed by ECL (ThermoFisher-Pierce, Rockford, IL, USA).

### 2.9. Statistical Analysis

Data were obtained from at least five independent experiments done in duplicate or triplicate. Unless indicated otherwise, arithmetic means ± standard deviations were calculated. The non-parametric Mann–Whitney test for median comparison was used when required (GraphPad Prism 6.0, GraphPad Software Inc., La Jolla, CA, USA); in all cases, *p* values < 0.005 were considered significant. Sample size was not statistically assessed and no randomization was done.

## 3. Results

### 3.1. Unprocessed Capsid of DENV2 Does Not Affect Viral Infectivity

**Figure 1:**
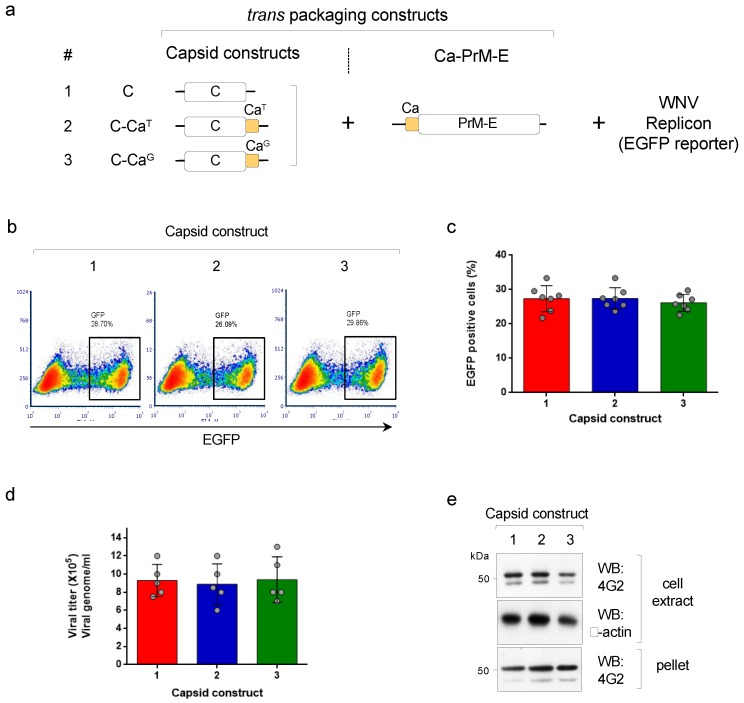
(**a**) Scheme of the tripartite system to produce pseudoviruses, showing the trans-packaging constructs encoding C or C-Ca (numbered 1–3) and the Ca-PrME and the WNV replicon (with EGFP reporter). (**b**) Representative cytofluorimetry (EGFP) of VERO cells infected with the pseudoviruses produced with the indicated capsid constructs. (**c**) Quantification of data shown in (**b**) (*N* = 7). (**d**) Quantification of viral genomes packaged in the indicated pseudovirus preparations (*N* = 5) (**e**) Western blot of cell extracts and pellet supernatants of cells producing the indicated pseudoviruses, developed with mAb 4G2 (anti-E). β-actin was used as the loading control.

The production of pseudoviruses using a WNV replicon (encoding the non-structural proteins and the EGFP reporter) and the DENV2 structural proteins can be used to understand the effect of providing an anchored capsid (C-Ca) on the assembly and infectivity of particles. We are hereby referring to C as the mature capsid protein cleaved from Ca. We first implemented a tripartite complementation approach, as previously described [15], that uses three transcription units: (i) a WNV replicon (that contains an EGFP reporter) and (ii) two trans-packaging constructs encoding, one, the structural proteins PrME and, the other, the capsid, either in the mature processed form (called soluble C) or the anchored, unprocessed form (C-Ca) (scheme shown in Figure 1a). Two versions of the anchored capsid C-Ca were used: one that was not cleaved by the WNV protease, where the first amino acid of Ca was Thr(T101), and one carrying the T101G mutation that was cleaved by the WNV protease (termed, respectively, C-Ca^T^ and C-Ca^G^ (Figure 1a). Production of pseudoviruses following co-transfection of the three components into HEK293T cells was then determined by cytofluorimetry of infected Vero cells (Figure 1b). The results of the infectivity assay were totally unexpected. To our surprise, all three constructs encoding either soluble C or C-Ca with T101 or T101G, produced comparable levels of infective DENV2 pseudoviral particles irrespective of whether Ca was or was not cleavable by the viral protease (Figure 1b,c). Furthermore, quantification of the viral RNA genome from the pelleted supernatant of the transfected cells, using 3’UTR specific primers, showed that all three variants—C, C-Ca^T^ and C-Ca^G^—were able to package the viral RNA without significant differences (Figure 1d). Western blot analysis of the intracellular and secreted levels of the protein E (detected with mAb 4G2) were in close correlation with the production of pseudoviral particles, further confirming the similar efficiency of the different capsid constructs (Figure 1e).

**Figure 2:**
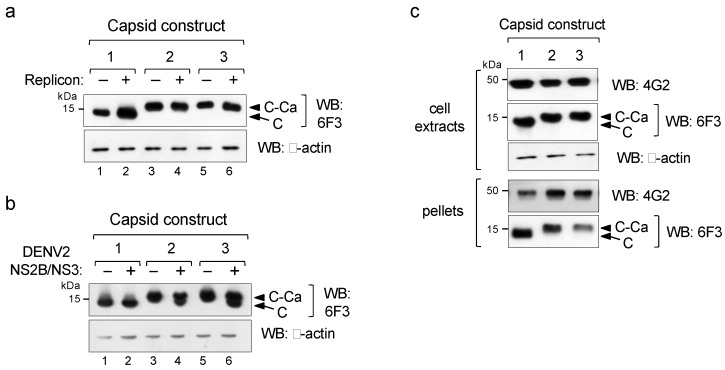
Processing by viral proteases. (**a**,**b**) Western blot of extracts of cells co-transfected with the indicated capsid constructs (shown in Figure 1) in the absence (−) or presence (+)of WNV replicon (**a**) or DENV2 NS2B/NS3 protease (**b**). (**c**) Western blot of cellular extracts and viral particles pelleted from culture supernatants (pellets) from cells producing pseudovirus particles, revealed with mAbs 4G2 for protein E and 6F3 for C, respectively. β-actinwas used as the loading control.

As the use of soluble (C) or anchored capsids (C-Ca) showed the same packaging efficiency and infectivity, we then investigated the cleavage efficiency of the C-Ca constructs by the WNV protease. Western blot analysis with the DENV2-specificanti-capsid mAb 6F3 showed no processing irrespective of the Ca peptide sequence, as only C-Ca was observed (Figure 2a, lanes 3–6). In contrast, when tested with the DENV2 NS2B/NS3 protease, both anchored C-Cas (C-Ca^T^ and C-Ca^G^) were processed, albeit not completely (Figure 2b). Because of this different processing efficiency, the WNV replicon system provides an excellent opportunity to investigate the effect of the unprocessed immature capsid on virus assembly and secretion.

We thus analysed whether unprocessed C-Ca was present in the infective pseudoviral particles secreted by the transfected cells. For this purpose, cells supernatants were ultra centrifuged through a sucrose cushion and the pelleted material was analysed by Western blot to detect the incorporated C and E proteins. Protein E was equally processed in all three total cell extracts as well as in the corresponding pseudoviral preparations (pellets) (Figure 2c). Conversely, samples from the two constructs expressing C-Ca (2 and 3) were found un-cleaved, both in the cell extracts and, more importantly, in the pseudovirus pellet fractions, thus indicating that these infectious particles were assembled with the un-cleaved C-Ca protein (Figure 2c).

### 3.2. Uncoupling Cleavage at the C-Ca-PrM Junction and Its Impact on Infective Particle Production

**Figure 3:**
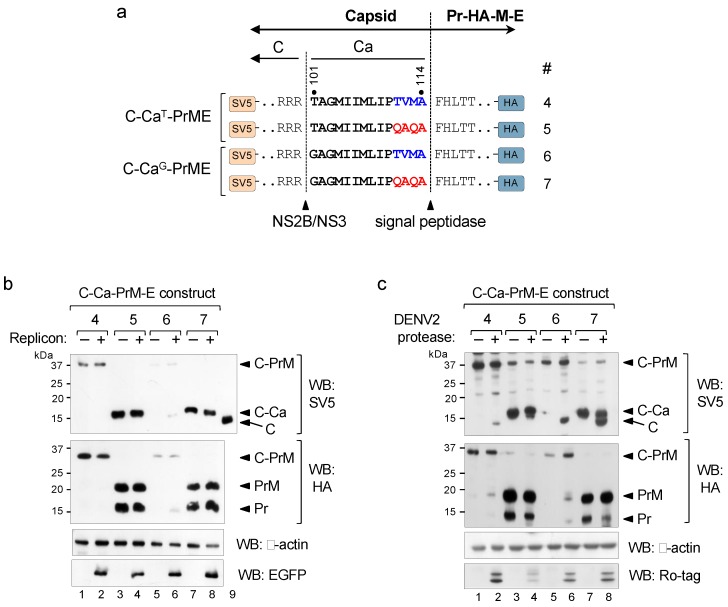
Uncoupling sequential processing. (**a**) Schematic representation of the C-Ca-PrME packaging constructs with wild-type (TVMA) or mutated (QAQA) Ca (numbered 4–7) and tagged with SV5 at the N-terminus of C and with HA at the C-terminus of Pr. Cleavage sites of NS2B/NS3 and signal peptidase are indicated. (**b**) Western blot of cellular extracts of cells co-transfected with the indicated constructs and with (+) or without (−) WNV replicon, revealed with anti-SV5 (upper panel) and with anti-HA (bottom panel). Replicon expression was controlled with anti-EGFP.Lane 9 corresponds to cells transfected with a construct encoding only C and included as a marker of migration of the mature C protein. Panel (**c**) is the same as (**b**) with (+) or without (−) DENV2 NS2B/NS3 protease. Expression of DENV2 NS2B/NS3 protease was controlled with anti-Ro_-_tag. β-actin was used as the loading control.

The results of the tripartite approach showed that the unprocessed C-Ca capsid was as efficient as the mature C in packaging of the RNA genome and, hence, in the formation of infective particles. We then examined whether the same was true in the bipartite system in which a single unit encodes the C-Ca-PrM-E polyprotein. As this required disruption of the sequential cleavage at the C-Ca-Pr junction, we introduced a substitution mutation at the C-terminus of Ca (from TVMA to QAQA) in the packaging constructs (scheme in Figure 3a). Such substitution was previously described to allow cleavage of other flavivirus Ca by the signal peptidase in the absence of cytosolic viral protease cleavage [14].These packaging constructs (4 to 7), containing either the wt (T101) or the T101G mutation, were tagged with SV5 at the N-terminus of C and with HA at the C-terminus of Pr just upstream of the furin site, to facilitate analysis of cleavage efficiency at the C-Ca-PrM junction by Western blots (Figure 3a).

In the absence of the viral protease (−WNV replicon) both QAQA mutants (constructs 5 and 7) were completely processed by the signal peptidase as only PrM and unprocessed C-Ca were observed (Figure 3b; construct 5 and 7), whereas constructs with the original Ca sequences (TVMA) showed the presence of only unprocessed forms (C-Ca-PrM) (Figure 3b; construct 4 and 6). In contrast, in the presence of the viral protease (+WNV replicon), as expected, construct 4 did not show any processing, and construct 6 was partially processed (Figure 3b, lanes 2 and 6). The two QAQA mutants instead, showed the same processing phenotype, i.e., unprocessed capsid (C-Ca) and processed PrM (Figure 3b, lanes 4 and 8). Taken together, these results indicate that the unprocessed capsid C-Ca generated following non-sequential cleavage is not a substrate for the WNV viral protease. Processing of aforementioned constructs with the DENV2 NS2B/NS3 protease showed that the QAQA mutants were only partially processed into mature C (Figure 3c, upper panel, lanes 7–8), similar to the phenotype observed with the tripartite system.

**Figure 4:**
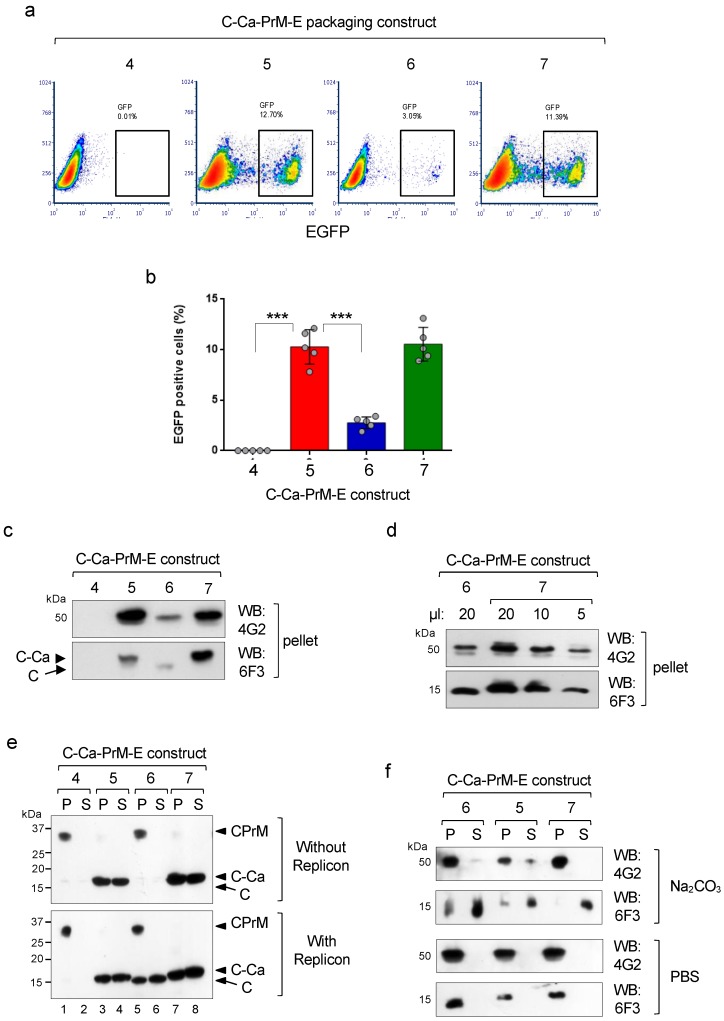
Effect of uncoupled sequential processing on viral infectivity. (**a**) Representative cytofluorimetry (EGFP) of VERO cells infected with the pseudoviruses produced with the indicated C-Ca-PrME packaging constructs. (**b**) Quantification of data shown in (**a**), (*N* = 5). (**c**) Western blot of particles produced with the indicated constructs and recovered by ultra centrifugation, and analysed for content of protein E (mAb 4G2) and C (mAb 6F3). (**d**) Relative quantification of particles recovered with constructs 6 and 7. (**e**) Western blot of cellular extracts of cells co-transfected with the indicated packaging constructs and without (upper panel) or with (bottom panel) WNV replicon and extracted with sodium carbonate. Supernatants (S, soluble fraction) and pellets (P, membrane associated fraction) were analysed with mAb 6F3 to reveal processed and unprocessed isoforms. (**f**) Western blot of particles obtained with the indicated constructs and extracted with sodium carbonate (upper panel) or with control PBS (bottom panel). The distribution of the two proteins E and C, in the two fractions (S, soluble and P, membrane associated) were analysed with mAbs 4G2 and 6F3, respectively.

To determine the effect of theTVMA to QAQA mutations on the production of infective pseudoviral particles, the untagged packaging constructs were co-transfected in HEK293T cells with the WNV replicon, and the infective particles in the supernatants were analysed by infection of Vero cells. As expected, only the Ca^G^ variant with the wild-type TVMA sequence(construct 6) that was partially processed was able to produce infective particles, while the unprocessed Ca^T^ (construct 4) was not (Figure 4a,b). Instead, the two QAQA mutants significantly enhanced production of infective particles in both the T101 and T101G Ca versions, despite the lack ofC-Ca processing by the WNV protease (Figure 4a,b). These results indicate that cleavage at the luminal side (but not at the cytosolic side) is determinant for assembly and secretion of DENV infective particles. In fact, viral particles from ultra centrifuged culture supernatants (over 20% sucrose cushion) analysed by immunoblotting showed correlation of the E and C levels with the relative infectivity (Figure 4c). Notably, unprocessed C-Ca (in constructs 5 and 7) was efficiently packaged by infective particles upon uncoupled processing, while construct 6 with Ca^G^ and with wild-type TVMA incorporated only mature C (Figure 4c). Furthermore, analysis of the relative number of secreted particles with construct 7, with respect to construct 6, was found to be approximately three times higher (Figure 4d).

As Ca is hydrophobic, we then investigated whether the unprocessed C-Ca product was attached or not to intracellular membranes. For this purpose, the tagged versions of the structural polyprotein were transfected in HEK293T cells in the absence or presence of the WNV protease. Cells were collected 36 hr post transfection, lysed in a hypotonic buffer and the lysate treated with sodium carbonate and ultra centrifuged to obtain the fractions of soluble (supernatants, S) and membrane-associated proteins (pellets, P). Western blot analysis of these fractions showed that the unprocessed C-Ca from the two constructs with the QAQA mutation was present in comparable amounts in the membrane and soluble fractions, irrespective of the presence or absence of the protease (constructs 5 and 7; Figure 4e). In contrast, all the material from the wild-type constructs (4 and 6), totally un-cleaved by the signal peptidase was present only in the membrane fraction in the absence of replicon (Figure 4e, upper panel). In the presence of replicon construct 6 (T101G) was partially processed on the luminal and cytosolic sides generating mature C, which was also similarly distributed in the two fractions (Figure 4e, bottom panel, lanes 5–6), whereas construct 4 (T101), as expected, remained unprocessed in the membrane fraction. These results suggest that the unprocessed C-Ca, despite its hydrophobic Ca domain, is, in part, soluble with a cellular localisation similar to mature C which was found as well only in part in the soluble fraction.

We then asked whether the unprocessed C-Ca incorporated into pseudoviral particles was anchored to the membrane envelope. For this, purified viral particles were treated with Na_2_CO_3_ and fractionated into the soluble and membrane fractions. The soluble fraction was concentrated by TCA precipitation. Western blot analysis showed that most of the protein E was present in the pellet as expected because of the two TM anchor domains, while most of the capsid, both mature C (construct 6) or unprocessed C-Ca (constructs 5 and 7) were found in the supernatant (Figure 4f). These results strongly suggest that the C-Ca protein incorporated into the infective particles was not strongly associated to the membrane envelope.

In conclusion, these results show that for DENV2 structural proteins, processing at the C-Ca boundary (namely, at the Ca N-terminus) by the viral protease is not a requisite for the assembly of infective particles.

## 4. Discussion

The flavivirus Ca plays the role of a signal sequence for PrM translocation into the ER lumen. Typical signal sequences have a low degree of sequence conservation but have common structural motifs: (i) an N-terminus rich in basic residues; (ii) a hydrophobic core; and iii) a C-terminus cleavage site, which must conform to the requirement of small residues such as alanine, in positions − and −3 upstream of the cleavage site and must be in an extended conformation [24,25,26]. Among flaviviruses, the carboxy-terminal part of Ca is atypically nonpolar. This feature prevents cleavage by the host signal peptidase in the absence of viral protease-mediated cleavage at the amino terminus [27,28].

This sequential temporal balance of the viral and host protease-mediated cleavages at the junction of C-Ca-PrM is considered a critical step in the biogenesis of flaviviruses [8,12,13,29,30]. Previous studies have shown that uncoupling of these cleavages by introducing mutations at the C-terminal region of Ca that allow efficient cleavage at the luminal PrM, renders C-Ca a poor substrate for the viral protease [13,14] and have detrimental effect on the production of infective particles [14,31]. In this work, we studied the involvement of these factors on processing ofthe DENV2 polyprotein and, hence, on the production of infective viral particles. For this purpose, we took advantage of the context dependent cleavage of the WNV protease which is unable to cleave DENV2 C-Ca because of the presence of a threonine residue at position P’1 [32].

To understand the effect of unprocessed C-Ca on the production and infectivity of DENV2 particles, we initially used a tripartite system for the production of infective pseudoviruses as previously described [15]. The DENV2 capsid constructs used in this study, namely, mature C or C-Ca with either wild-type (T101) or mutated Ca (T101G) showed comparable efficiency in the production of infective pseudoviral particles, unlike other flaviviruses reported to have diminished effect on viral production in similar assays [14,31]. The most surprising result, however, was obtained with the wild-type C-Caconstruct, which was not cleaved by the WNV protease and still produced infective particles. From these results, we expected that the absence of downstream PrME might affect the steric constraint at the C-Ca junction created by the Thr101 residue and, hence, processed by the WNV protease. In contrast to our hypothesis, the cleavage analysis showed that C-Ca is not a substrate for WNV protease in the absence of the downstream polyprotein, irrespective of the residue at the protease cleavage site. Unlike the WNV protease, the DENV2 protease could partially process C-Ca, generating mature capsid.

Previous studies have shown that the mature C protein of flaviviruses localise to different cellular compartments like lipid droplets and nucleolus of the infected cells [33,34,35,36,37]. Our results indicate that the unprocessed C-Ca had a similar behaviour as the mature protein. Analysis of the ultra centrifuged pseudoviruses showed, in addition, that C-Ca was incorporated into the infective particles.

We also used a bipartite approach to obtain pseudoviruses, in which the sequential cleavage of Ca was disrupted by a substitution mutation (i.e., QAQA at the C-terminus of the Ca sequence). This mutation, known to favour cleavage by the signal peptidase irrespective of the viral protease cleavage [14], showed the generation of mature PrM and unprocessed C-Ca, thus confirming that C-Ca is not a suitable substrate for the WNV viral protease in the absence of the sequential processing while still able to be packaged into pseudoviral infective particles. These results were unprecedented, as these mutants produced more infective particles compared to packaging construct with typical Ca sequences.

To date, the presence of C-Ca has been considered to have an inhibitory effect on virion assembly. Unlike DENV2, other flaviviruses like MVEV and YFV showed contrasting phenotypes upon disturbance in the sequential cleavage. These viruses were not able to incorporate C-Ca [12,13,14,30]. It is possible that C-Ca, in the absence of the downstream PrM protein (as in the tripartite system), does not get anchored to the ER membrane and behaves like the cleaved mature C. We showed evidence, with the carbonate extraction assay, that this was indeed the case. The carbonate extraction of membrane bound and soluble fractions from cell extracts showed similar profile in the case of both mature C and unprocessed C-Ca. Also, in the secreted particles, most of C and C-Ca was found in the soluble fraction. For C-Ca, it is possible that the limited Ca length as well as the QAQA mutation, which is less hydrophobic than the original TVMA, play a role in preventing a strong association to the membrane envelope.

These discrepancies between our study and those on other flaviviruses entail virus-specific differences in the accessibility of the C-Ca cleavage site to the viral protease and its incorporation into infective particles. We hypothesised that the reason for these differences between DENV and other flaviviruses could be due to the exceptionally shorter Ca region which might not be long enough to anchor C-Ca to the ER membrane, unlike viruses with longer Ca lengths (ranging from 18–22 amino acids). Schrauf and co-authors [38] have shown that the C terminus of YFV and WNV C protein can accommodate some extensions (like the 2A peptide) that eliminate the requirement for viral protease cleavage without affecting the formation of infectious viral particles.

Overall, our results indicate that in mammalian cells, incorporation of C-Ca in DENV2 pseudoviral infective particles is permissive with no adverse effects. It also demonstrates that while processing at the luminal side by the signal peptidase is crucial for particles assembly, the same is not true for cleavage at the cytosolic side. These results suggest that Ca sequential processing might have been acquired during viral evolution from ancestor precursors able to incorporate the anchored C-Ca protein. Future studies could address whether our findings in mammalian cells can also take place in mosquitoes.
